# Divide-and-Conquer Matrisome Protein (DC-MaP) Strategy: An MS-Friendly Approach to Proteomic Matrisome Characterization

**DOI:** 10.3390/ijms21239141

**Published:** 2020-11-30

**Authors:** Emna Ouni, Sébastien Pyr dit Ruys, Marie-Madeleine Dolmans, Gaëtan Herinckx, Didier Vertommen, Christiani A. Amorim

**Affiliations:** 1 Pôle de Recherche en Gynécologie, Institut de Recherche Expérimentale et Clinique, Université Catholique de Louvain, 1200 Brussels, Belgium; emna.ouni@uclouvain.be (E.O.); marie-madeleine.dolmans@uclouvain.be (M.-M.D.); 2 de Duve Institute, Université Catholique de Louvain, 1200 Brussels, Belgium; sebastien.pyrditruys@uclouvain.be (S.P.d.R.); gaetan.herinckx@uclouvain.be (G.H.); didier.vertommen@uclouvain.be (D.V.); 3 Gynecology and Andrology Department, Cliniques Universitaires Saint-Luc, 1200 Brussels, Belgium

**Keywords:** ECM, proteomics, ovary

## Abstract

Currently, the extracellular matrix (ECM) is considered a pivotal complex meshwork of macromolecules playing a plethora of biomolecular functions in health and disease beyond its commonly known mechanical role. Only by unraveling its composition can we leverage related tissue engineering and pharmacological efforts. Nevertheless, its unbiased proteomic identification still encounters some limitations mainly due to partial ECM enrichment by precipitation, sequential fractionation using unfriendly-mass spectrometry (MS) detergents, and resuspension with harsh reagents that need to be entirely removed prior to analysis. These methods can be technically challenging and labor-intensive, which affects the reproducibility of ECM identification and induces protein loss. Here, we present a simple new method applicable to tissue fragments of 10 mg and more. The technique has been validated on human ovarian tissue and involves a standardized procedure for sample processing with an MS-compatible detergent and combined centrifugation. This two-step protocol eliminates the need for laborious sample clarification and divides our samples into 2 fractions, soluble and insoluble, successively enriched with matrisome-associated (ECM-interacting) and core matrisome (structural ECM) proteins.

## 1. Introduction

Identification and quantification of the components of distinct extracellular matrix (ECM) proteins and comprehension of their underpinning network interactions represent key steps towards elucidating the role of the ECM in health and pathological conditions [1,2]. Proteomics offers an opportunity to address these challenges in a global and unbiased manner, without the need for hypothesis-driven molecular candidates. Indeed, the past decade has seen the emergence of mass spectrometry (MS)-based proteomics [3] as the method of choice to characterize the protein composition of the ECM [4,5,6,7,8].

Despite the significant development in data acquisition and identification of post-translational ECM modifications [9,10,11,12,13,14], extracellular proteins still present analytical difficulties in proteomic strategies due to the biochemical characteristics of these molecules, which are highly cross-linked, often glycosylated and hence insoluble even in strong detergents and organic denaturants [8]. Obtaining the fullest picture of ECM protein composition is still hampered by its complex biochemical nature and wide dynamic range. Nevertheless, in recent years, different techniques have been proposed in an attempt to complete our understanding of the human matrisome in health and disease [2]. The matrisome is a contemporary extended definition of the ECM and is composed of 2 major protein categories: (i) core matrisome proteins, including ECM glycoproteins, collagens and proteoglycans; and (ii) ECM-associated proteins, consisting of ECM-affiliated proteins that show biochemical and architectural analogy with the ECM or are known to be associated, ECM regulators that are proteins responsible for ECM turnover, and secreted factors that interact with core ECM proteins [8].

Since only the most abundant proteins are identified in non-targeted proteomics, the ability to selectively enrich for ECM proteins is the first key step of dedicated proteomic pipelines. Recent papers have reported the use of buffers with defined salt and detergent concentrations and increased stringency to sequentially extract (or fully deplete) intracellular components and thereby enrich for less soluble components, mainly core matrisome proteins, and improve ECM-associated protein detection [15]. In this context, ionic, non-ionic, and zwitterionic detergents have been used to solubilize ECM-enriched samples [16]. However, their use in MS presents some limitations. Although SDS is the most widely used detergent [17,18,19], even at low concentrations (0.01%) it can severely suppress the ionization of proteins/peptides and hamper their identification. Non-ionic surfactants mostly considered as mild surfactants such as Triton X-100 and NP-40 can cause significant signal suppression and high polymer background in addition to the formation of adducts with the proteins [20]. Zwitterionic surfactants such as CHAPS, characterized by intermediate protein solubilizing abilities, being stronger than non-ionic detergents but not as strong as ionic surfactants (e.g., SDS) have been also used in MS, despite causing adduct formation and signal suppression [20].

As an alternative to detergents, resuspension of ECM-enriched protein samples prior to digestion can also be achieved by the use of high concentrations of chaotropic agents such as urea or guanidinium chloride (GdnHCl) [21] and concomitant use of reducing agents. The currently accepted and widely used solubilization method to obtain ECM-enriched pellets for bottom-up proteomic analysis relies on two reagents: cyanogen bromide (CNBr), which cleaves peptide bonds at the C-terminus of methionine [22]; and hydroxylamine (NH_2_OH), which cleaves at the C-terminus of asparagine and the N-terminus of glycine [2,21]. Comparison of these two non-enzymatic cleavage approaches by Hansen’s group revealed that hydroxylamine reduced analytical variability compared to CNBr digestion and avoided CNBr toxicity and labor-intensive handling [21,23]. While the NH_2_OH method yielded higher concentrations of ECM components and more reproducible measures between replicates, the cleavage specificity of the two chemical approaches presented some limitations that needed further consideration when conducting database searches [21]. The authors also mentioned that quantification of peptides containing asparagine residues should be performed with caution since the NH_2_OH cleavage site is not strictly asparagine-specific [21].

Despite some success in overcoming solubility issues of ECM molecules, dialysis, gel filtration, or filter-aided sample preparation (FASP) are required before protein digestion and MS detection for removal of excess detergents, salts and chemicals [18]. Unfortunately, these processes of sample clarification usually induce significant protein sample loss due to buffer exchange and redissolving steps, compromising the sensitivity, reliability and accuracy of proteomic analysis and, more specifically, identification of matrisome composition [24]. With only micrograms of samples available from human biopsies, fractionation becomes an even greater challenge. Successive sample loss can be crippling, and dilution of highly complex mixtures can lead to a deficit of low-abundance protein species below signal-to-noise thresholds.

Decellularization offers another option to enrich ECM proteins while removing intracellular material. However, complete decellularization requires several hours to days of extensive physical and chemical treatment. This may lead to protein degradation and loss of matrisome-associated proteins due to buffer exchanges during the whole process, which could in turn cause poor reproducibility and low recovery [25].

Recently, MS-compatible surfactants have been developed to overcome common limitations of standard detergents described above, namely RapiGest (RP), ProteaseMax (PM), and 3273 (also known as MaSDeS) [16,26,27]. These molecules contain an acid-labile functional group, which quickly degrades into innocuous non-surfactant byproducts upon addition of acid, eliminating the need to remove the detergent prior to MS analysis (effectively rendering it MS-compatible). In addition to these acid-labile surfactants, non-ionic saccharide surfactants such as n-dodecyl β-D-maltoside (DDM) are described as MS-compatible at relatively low concentrations and have been applied for many years for insoluble membranous protein characterization [28,29], but they have never been used for matrisome characterization.

Here, we describe the first application of MS-compatible detergents in matrisome proteomics and propose a novel method of optimized tissue sampling and mechanical digestion adapted to small biological samples before initiating a fractionated extraction. Our protocol is tailored to matrisome proteomics to overcome current limitations in the field and provide a reliable screening, not only of the structural ECM, but also of associated proteins able to regulate and remodel the ECM, through a simple and reproducible workflow.

## 2. Results

### 2.1. Stainless Steel Beads Allow Optimal Mechanical Lysis

**Figure 1:**
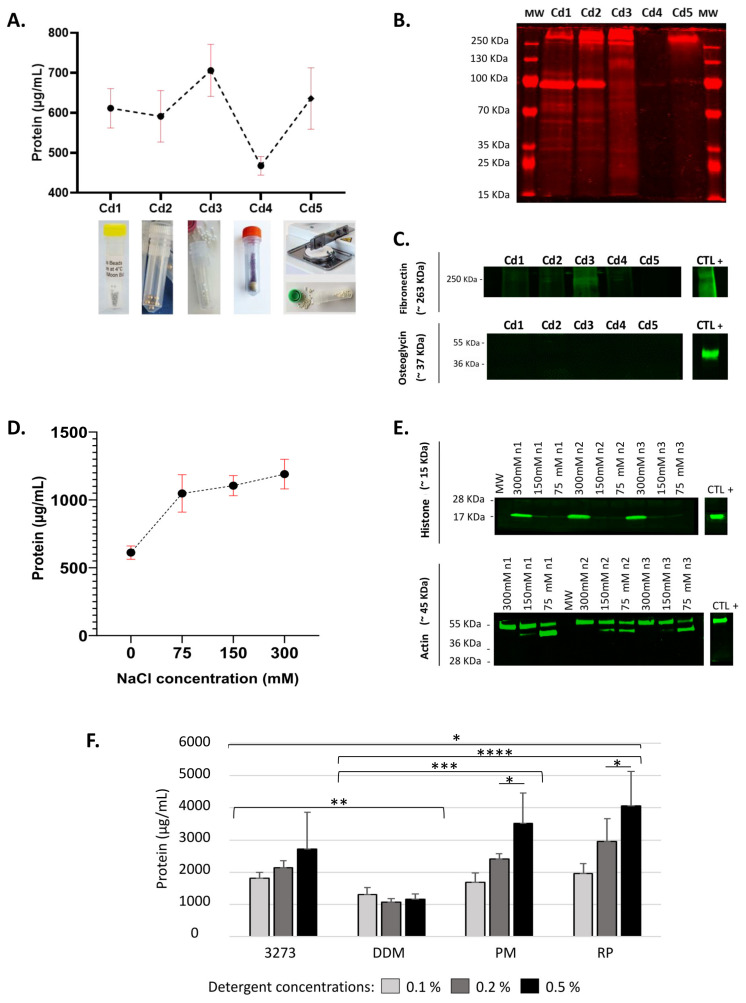
Choice of mechanical lysing matrix and buffer formulation. (**A**) Comparison of 5 lysis beads (*n* = 3) based on the total amount of soluble extracted proteins (± SD, red lines) from 10 mg samples. The size and nature of lysis matrices is described as follow: Cd1, 0.3 mm stainless steel beads; Cd2, 2.8 mm stainless steel beads; Cd3, 0.5 mm zirconium oxide beads; Cd4, a garnit matrix with a 6.3 mm ceramic sphere; and Cd5, 1.4 mm ceramic beads. Twenty micrograms of total proteins and a molecular weight marker (MW) were loaded per gel, (**B**) their quality evaluated by electrophoresis, and (**C**) preservation of core matrisome proteins was estimated by Western blot. Ovarian tissue homogenate was used as a positive control (CTL+). (**D**) Three concentrations of NaCl were used to enrich HEPES lysis buffer (*n* = 3): 75 mM, 150 mM and 300 mM with Cd1 lysis beads. Their evaluation was based on the total amount of extracted proteins (± SD, red lines) and (**E**) their cellular compartment (histone, nucleus; actin, cytoskeleton) in all biological replicates (n1, n2, n3). (**F**) Protein yield obtained by use of Cd1 as a lysing matrix, and 25 mM HEPES and 300 mM NaCl as the optimal lysis buffer to resuspend mass spectrometry-compatible detergents. Two-way ANOVA was applied to compare detergent performance independently of the concentration used, before comparing outcomes with the same detergent at different concentrations (* *p* 0.05, ** *p* 0.01, *** *p* 0.001, **** *p* 0.0001).

Ovarian tissue is inherently mechanically resilient. Therefore, to successively homogenize our samples, we chose to apply a mechanical disruption method using 5 different commercial lysing matrices, which yielded different amounts of proteins (Figure 1A). None of the lysing matrices tested left behind any tissue clumps. Based on total extracted protein amounts, we could eliminate condition Cd4, a garnet matrix with one 6.3 mm ceramic sphere that extracted the lowest amount of proteins with a muddy homogenate aspect. While the Cd5 lysing matrix, 1.4 mm ceramic beads delivered promising outcomes in terms of total protein amounts, it showed high variability explained by the more elaborate and time-consuming two-step process: (i) tissue chopping and (ii) disruption of the tissue clumps with 1.4 mm ceramic spheres. Cd3, 0.5 mm zirconium oxide beads extracted the highest protein yield, but the quality profile visualized by total protein labeling on SDS-PAGE revealed poor protein quality (faint and diffuse bands) affected by the mechanical disruption (Figure 1B). Immunoblotting was conducted on supernatants of the pass-throughs, which are composed of soluble proteins, in order to detect any potential impact of lysing matrices on ECM protein solubilization. We observed ECM solubilization under the influence of Cd3, highlighted by a high detected level of fibronectin in the soluble fraction (fraction 1) (Figure 1C). This could later harm the fractionation process and impact the dynamic range.

Two options were left: Cd1 and Cd2. Cd2, 2.8 mm stainless steel beads occasionally led to the fracture of the lysis tube during homogenization and loss of samples, but Cd1 yielded more reproducible results and ease of handling. In addition, it showed a better protein quality profile and good preservation of ECM proteins in the insoluble fraction (fraction 2), highlighted by weak band intensity of fibronectin and undetected osteoglycin (OGN, aka mimecan) in fraction 1 (Figure 1B,C). Thus, Cd1, composed of stainless steel beads measuring 0.3 mm in diameter, enabled optimal mechanical lysis of human ovarian tissue and was therefore used in all downstream procedures.

### 2.2. Potentialization of Protein Extraction Using 300 mM NaCl

Using Cd1 lysis beads, we investigated the extraction efficiency of HEPES lysis buffer containing different NaCl concentrations. Controlling salt concentrations in protein extraction buffers has a great impact on protein extraction yields [30,31]. Concentrations were carefully selected to enhance protein salting without rendering the residual pellet in fraction 2 more insoluble. Indeed, at low salt concentrations (0.5 M), protein solubility increases along with ionic strength [32].

Higher salt concentrations extracted more soluble proteins (Figure 1D). Primarily, we noted an increase of total protein amount of around 2-fold with 300 mM NaCl yielding the highest amount of extracted proteins. Western blot of histone H3 and actin revealed a difference in extraction of nuclear and cytoskeleton proteins among tested concentrations (Figure 1E). A 300 mM NaCl concentration enabled better extraction of nuclear and cytoskeleton proteins than all other conditions.

A final lysis buffer containing 300 mM NaCl in HEPES was therefore used to resuspend MS-compatible detergents. Quantification of soluble proteins in fraction 1 revealed that RP enabled the best extraction, showing a significantly higher yield with all 3 concentrations (0.1%, 0.2% and 0.5%) than 3273 (*p* 0.05) or DDM (*p* 0.00001) (Figure 1F). DDM delivered the poorest protein extraction, with no visible difference between tested concentrations. Most importantly, following addition of RP to the lysis buffer, we succeeded to obtain higher amounts of extracted proteins than with NaCl buffer alone, reaching 4000 µg/mL from 10 mg wet weight tissue.

### 2.3. Differential Protein Enrichment Favors Matrisome-Associated Protein Detection

**Figure 2:**
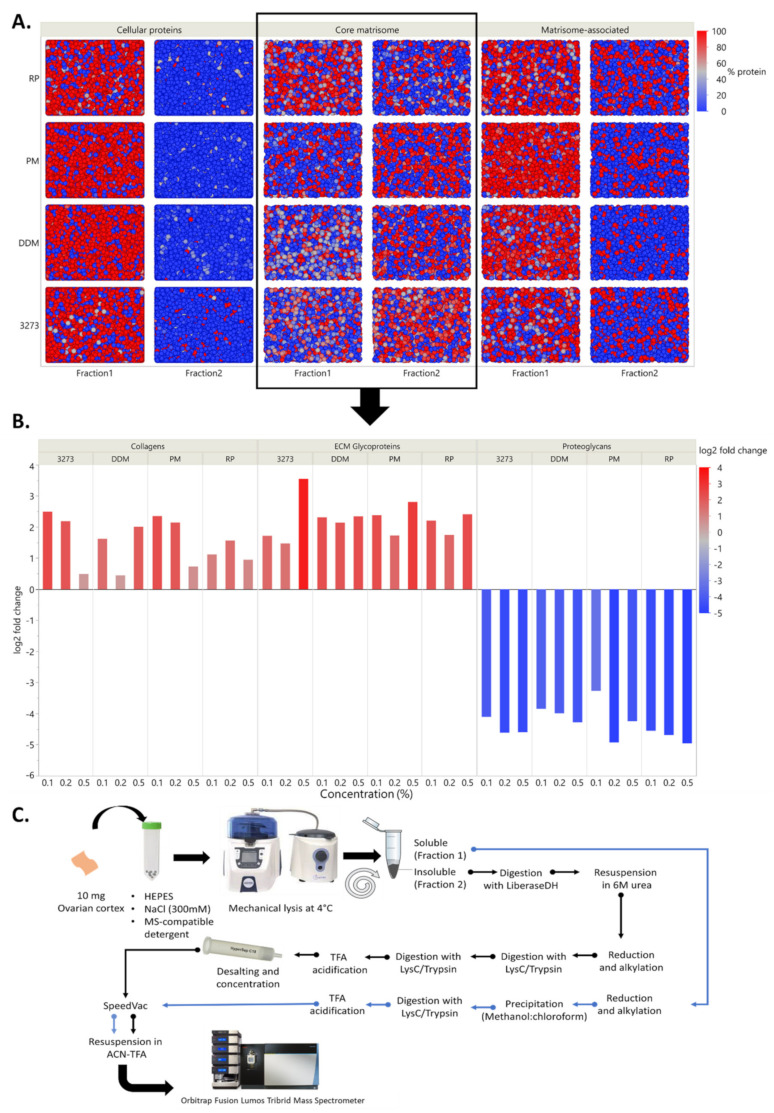
Two-step fractionation impact on the segregation of cellular protein, core matrisome and matrisome-associated proteins. (**A**) Scatter plot of proteomic data according to the protein percentage in each fraction; each dot represents a protein and the color key reflects its relative abundance per fraction. Matrisome data (framed) have been explored deeper in B. (**B**) Enrichment of collagen and glycoproteins in all conditions in fraction 2 expressed as the log2 fold change in XICs. (**C**) Summary of the two-step fractionation approach (DC-MaP technique).

**Figure 3:**
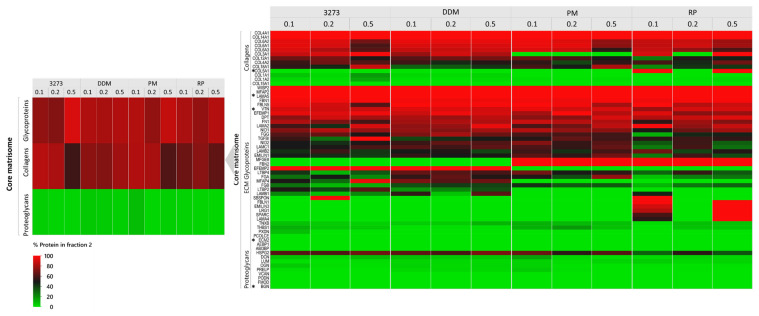

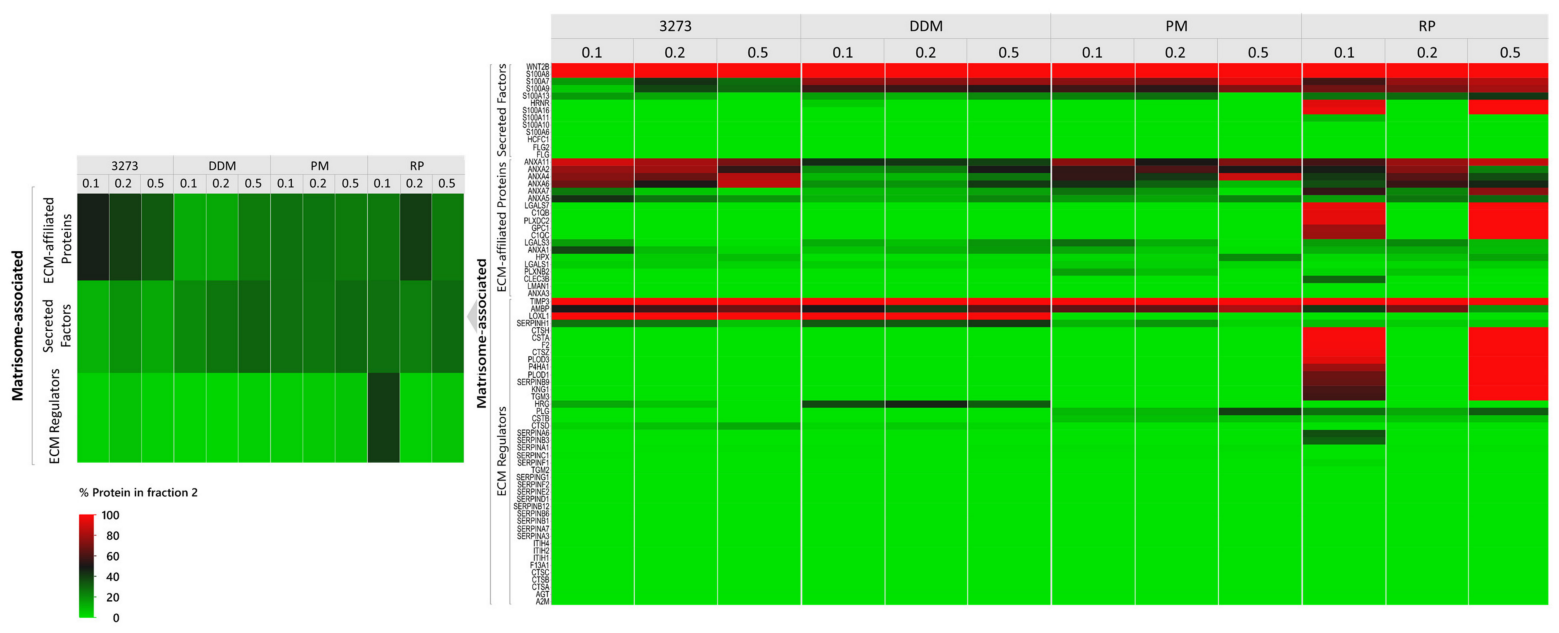
Heatmap of matrisome proteins detected in each condition. Protein abundance is expressed as the protein percentage detected in fraction 2 for each matrisome protein (**right side**) and averaged per category (**left side**). Proteins cited in the text are labeled with asterisks.

Our MS-compatible detergents, 3273, DDM, RP, and PM, were resuspended in the lysis buffer to achieve 0.1%, 0.2%, and 0.5% (*w*/*v*) concentrations and used during the mechanical lysis step to homogenize ovarian tissue. The obtained homogenate was centrifuged to recover the soluble supernatant labeled as “fraction 1” and the remaining pellet labeled as “fraction 2”. To be able to evaluate our fractionation procedure and follow the distribution of each protein between the two fractions, we assumed total amounts of each protein present within the same tissue sample to be equal to the sum of its normalized abundance (sampling rate normalization) in fractions 1 and 2, both expressed in peak integration of extracted ion chromatograms (XICs, area under the curve). To facilitate interpretation, we duly converted the data into percentages to evaluate how much of the same protein was extracted in fraction 1 and how much remained in fraction 2. By plotting all the quantitative data of each protein detected per condition, we were able to observe the ability of this procedure to isolate the majority of cellular proteins in fraction 1, as well as matrisome-associated proteins (Figure 2A), which are known to be soluble. Fraction 2 (the insoluble fraction), on the other hand, was more enriched in glycoproteins and collagens (Figure 2B). Such fractionation allowed better detection of low-abundance matrisome-associated proteins and reduced the dynamic range. Indeed, the total number of spotted matrisome-associated proteins reached 73 ± 2 (Figure 3 and Supplementary Table S1), among which 41 ± 1 ECM regulators, 19 ± 1 ECM affiliated proteins as well as 13 ± 0 secreted factors were confidently detected in all conditions, but with different detection levels in XICs.

By adopting 2-step fractionation using MS-compatible detergents (Figure 2C), we noted that 0.2% 3273 yielded the best matrisome protein enrichment in fraction 2 (70% enrichment of proteoglycans and 80% enrichment of ECM regulators and secreted factors) (Figure 3). Nevertheless, optimal enrichment of all matrisome proteins in just one fraction was not attainable, since in all conditions, some ECM proteins were 100% present in fraction 1 (BGN, ECM 2 and COL5A1), while others were 100% in fraction 2 (TIMP3, LAMA5, and VTC) (Figure 3 and Supplementary Data S1). To overcome this unavoidable limitation, we chose to combine matrisome protein detection in fractions 1 and 2 during post-acquisition, which revealed promising ECM protein discovery with 3273 and RP (Supplementary Datas S1 and S2). Overall, RP yielded a better identification than 3273 of core matrisome proteins, such as FBN2, an important elastic ECM protein that regulates the early process of elastic fiber assembly and has not been detected before in human premenopausal or menopausal ovaries. RP allowed better detection of MFGE8, another core matrisome protein that plays a pivotal role in the human ovary by mediating the regression process of atretic ovarian follicles [33]. With RP, detection of FBN2 and MFGE8 was 2-fold higher (based on log10 [quantile normalized abundance]) than with 3273 (Supplementary Data S3).

Matrisome-associated protein detection is quite challenging due to the high solubility of this category of proteins, which are embedded within the highly abundant core matrisome components (like collagen) that hamper their detection. Nevertheless, detection was improved with RP compared to 3273, where we saw cathepsins that participate in ECM degradation and turnover [34]. In this protein family, C1QC, C1QB, and CTSH were identified twice as often with RP (Supplementary Data S3). Moreover, among secreted factors, RP enabled greater identification of S100A16, a member of the serpin family, which plays an important role in ovarian function [35,36,37] and has prognostic value in ovarian cancer [38]. Conversely, 3273 produced a slightly higher collagen yield at all tested concentrations mainly, COL6A1/A2/A3 and COL12A1 (Supplementary Data S3). Nevertheless, all collagens identified with 3273 were also identified with RP, so 2-step fractionation using RP is the most sensitive method for matrisome protein identification, especially at 0.2% and 0.5% when summing the XICs of both fractions during post-acquisition. On the other hand, fractionation enhanced detection of low-abundance soluble proteins in fraction 1 by sending glycoproteins and collagens to fraction 2 (Figure 2B). Consequently, combining the results obtained from both fractions as described above enabled to capture the full picture of the ovarian matrisome (Figure 3, Supplementary Data S3).

### 2.4. Highest Reproducibility at 0.2% Detergent Concentration

**Figure 4:**
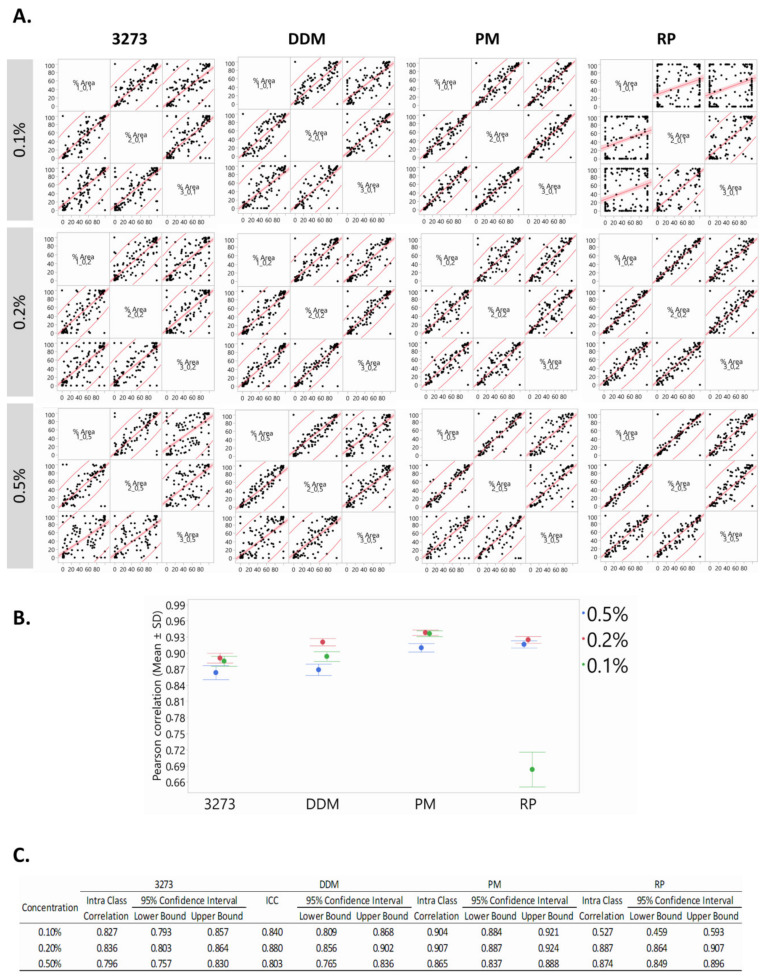
Reproducibility evaluation. (**A**) All biological replicates in each condition were compared and correlated two by two using Pearson’s correlation. (**B**) Plot of means of correlation coefficients calculated for each detergent at the 3 tested concentrations: 0.1%, 0.2%, and 0.5% (mean ± SD). (**C**) The intraclass correlation coefficient (ICC) was calculated per condition to further assess the consistency of each method, which proved the reliability of RP at 0.2%.

To obtain a reliable readout of the ovarian matrisome, reproducibility of protein identification is essential for clinical research and tissue engineering. Therefore, in order to select the most reproducible method, we used Pearson’s correlation between biological replicates in each condition (Figure 4). At 0.1%, correlation coefficient r of RP was the weakest relative to the rest of the tested conditions, but it reached a peak at 0.2% (Figure 4A,B). It is worth mentioning that at 0.5%, 3273 became more viscous, which could account for its relatively weak reproducibility at this concentration. Similarly, the highest tested concentration of DDM and RP gave the least reproducible outcomes. These results are further reflected in intraclass correlation coefficients (ICCs), and their delimited 95% confidence interval confirms the consistency of our methods (Figure 4C). ICCs assess the extent to which measurements can be replicated by reflecting not only the degree of correlation between replicates, but also agreement between measurements. This test was therefore conducted between replicates to further evaluate the reproducibility of each method. ICCs are measured on a scale of 0 to 1; 1 represents perfect consistency (reproducibility) with no measurement/technical error, while 0 indicates no reliability.

Considering sensitivity (defined as high qualitative and quantitative detection of matrisome proteins) in association with high reproducibility, 0.2% RP outperformed all the other MS-compatible detergents.

### 2.5. Optimal RP Performance in the Presence of Salt and Minimal Sample-Surface Contact

**Figure 5:**
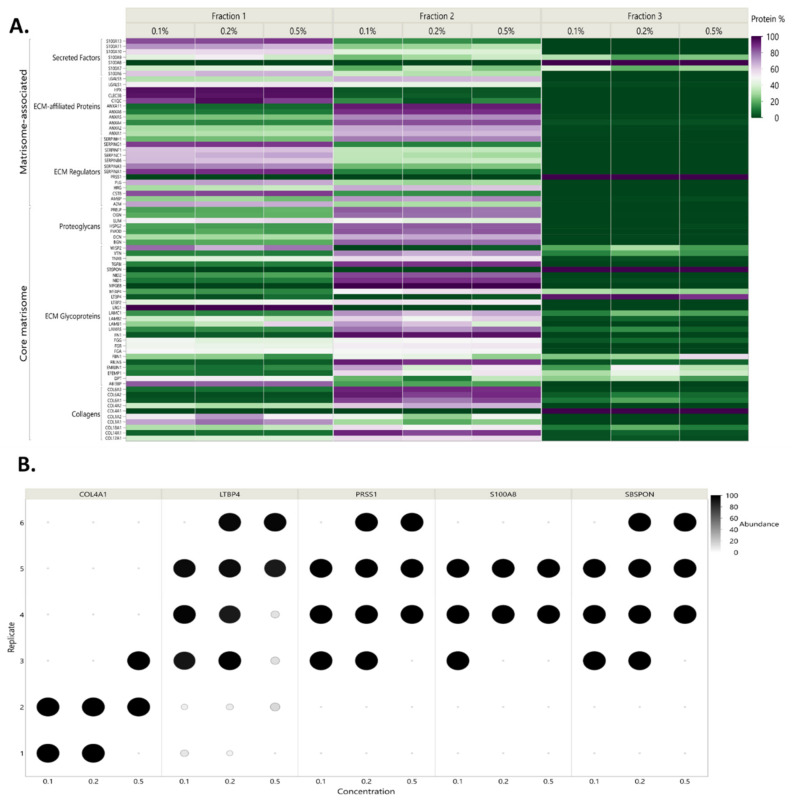

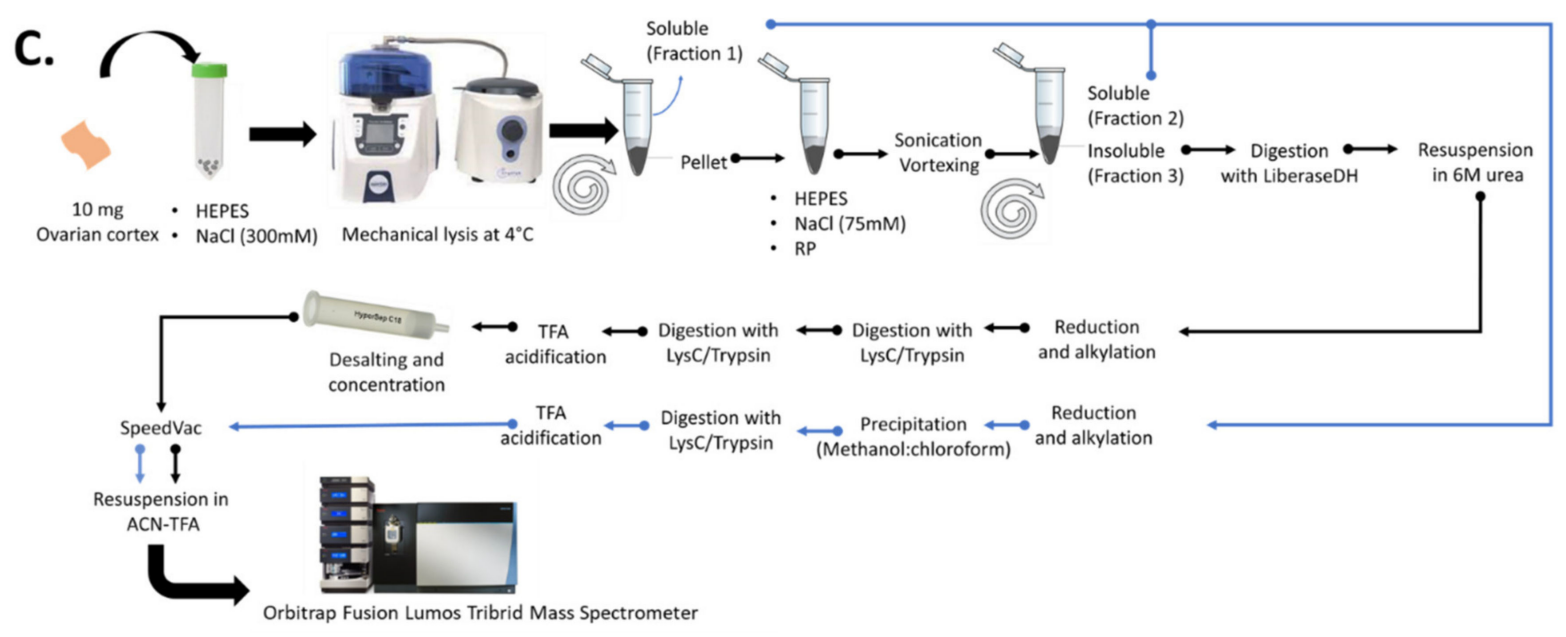
Three-step fractionation procedure outcomes. (**A**) Heatmap of matrisome protein abundance within the 3 obtained fractions highlights the localization of the majority of identified proteins in fractions 1 and 2. (**B**) Some proteins were exclusively detected in fraction 3, but their detection (abundance as a percentage in fraction 3) was very variable between the 6 tested replicates, as shown by the color and size of markers. (**C**) Summary of the three-step fractionation approach.

Once RP was selected as the most reliable MS-compatible detergent, we chose to add a third fractionation step to exclude the possibility of interference of high salt (NaCl) levels in the lysis buffer with the action of the surfactant and evaluate its impact on ECM detection (Figure 5). While 2-step fractionation starts with mechanical lysis in lysis buffer containing 300 mM NaCl and MS-compatible detergent, the 3-step procedure starts with mechanical lysis in the lysis buffer free of any detergents, and only the pellet is treated with MS-compatible detergent in a low-concentration NaCl buffer.

**Figure 6:**
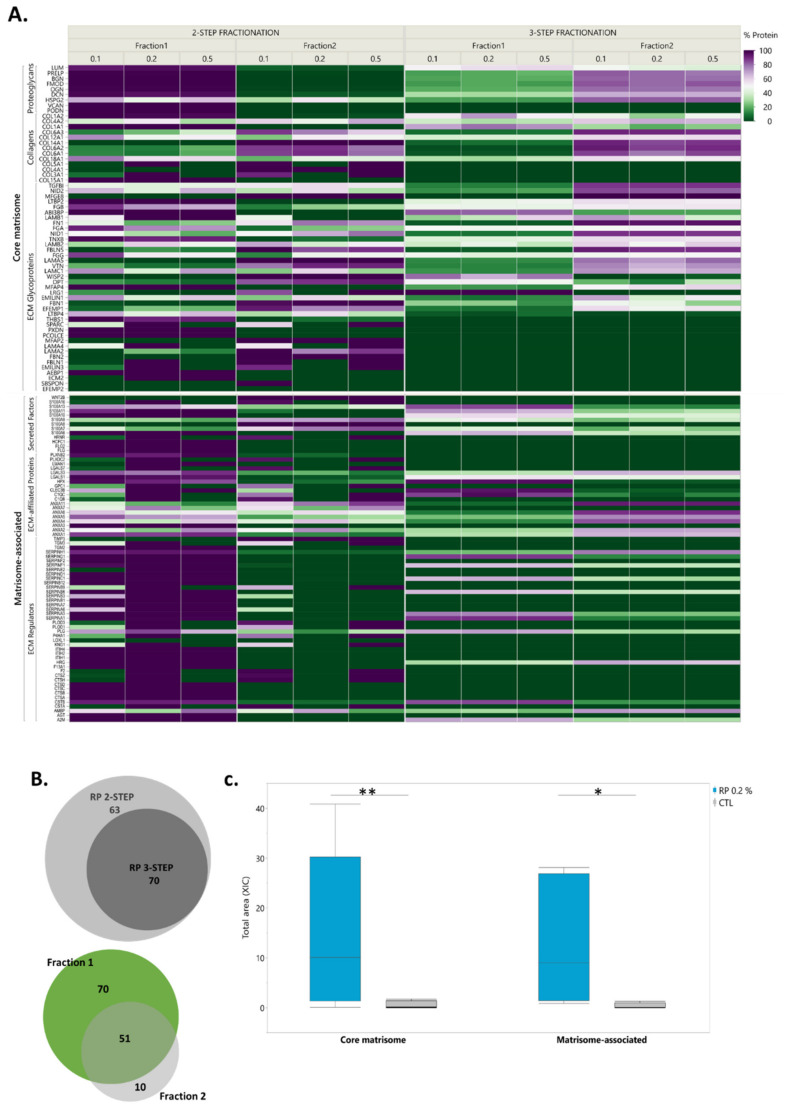
Superiority of the DC-MaP technique. (**A**) Comparison of the DC-MaP technique with the 3-step fractionation approach. Protein abundance is expressed as a percentage of each protein per fraction in each condition (**B**) Venn diagrams show unique and shared identified matrisome proteins between fractions 1 (soluble) and 2 (insoluble) using the DC-MaP technique and between both fractionation approaches using 0.2% RP. (**C**) Box plots of core matrisome and matrisome-associated protein levels identified with DC-MaP and a non-fractionated sample (CTL). A paired t-test and Wilcoxon’s test were used to compare the abundance of core matrisome and matrisome-associated proteins respectively (* *p* 0.05, ** *p* 0.01).

Contrary to our assumptions, RP action appeared to be optimal in contact with NaCl at 300 mM. Although 3 fractions were recovered in the 3-step procedure, only fraction 1 (HEPES pH 7.6, 300 mM NaCl) and fraction 2 (HEPES pH 7.6, 75 mM, RP) could accommodate the total amount of identified matrisome proteins (Figure 5A). The only proteins 100% present in fraction 3 (COL4A1, LTBP4, PRSS1, S100A8, and SBSPON) showed high variance between biological replicates (Figure 5B). Comparison of 2-step and 3-step fractionation also aimed to evaluate the possibility of further improving the dynamic range and washing away cellular proteins from ECM-containing fractions. Nevertheless, the results revealed the solubility of portions of matrisome proteins following mechanical lysis, even in HEPES with 75 mM NaCl, exposing the limitations of 3-step fractionation (Figure 6A). We observed that an increase in sample processing steps and surfaces harmed protein recovery and particularly matrisome proteins.

Indeed, this method includes vigorous agitation and long vortexing, which causes protein adsorption and loss with increased exposure to surfaces. The 2-step fractionation method enabled the exclusive identification of 63 matrisome proteins in addition to the detection of all proteins identified with the 3-step method (Figure 6B, Supplementary Data S3).

Hence, the 2-step fractionation procedure using 0.2% RP in the presence of 300 mM NaCl is the most tailored approach to matrisome proteomics.

### 2.6. To Fractionate or Not to Fractionate

To comprehensively characterize the human ovarian ECM, we previously developed a proteomic method with no centrifugal fractionation using two-dimensional liquid chromatography mass spectrometry (2D-LC/MS). This method, based on in silico matrisome identification, allowed confident identification of more than 80 matrisome proteins from a bulk of cellular proteins [39]. It was therefore essential to compare this technique with the current 2-step fractionation method using 0.2% RP in the same MS conditions (Figure 6B,C). The results revealed an 8-fold increase in the detected number of matrisome proteins, with around a 15-fold upturn in core matrisome abundance and 13-fold increase in matrisome-associated proteins. Thus, our new technique of 2-step fractionation using 0.2% RP clearly yields superior extraction and identification of ovarian matrisome proteins without the need to go through time-consuming 2D-LC/MS analysis.

## 3. Discussion

In our study, we set out to overcome the major challenges hampering our ability to accurately characterize the matrisome: MS-unfriendly detergents, irreproducibility, labor-intensive processing, and consequent loss of low-abundance proteins. To this end, we used the non-ionic saccharide surfactant DDM and acid-labile detergents 3273, RP, and PM, which are MS-friendly reagents that have similar properties to conventional detergents [40] and have been described as trypsin enhancers [41]. Cleavable detergents have the added benefit of quickly degrading into innocuous non-surfactant by-products when they are no longer required upon addition of acid, eliminating the need to remove them prior to MS analysis. However, these surfactants have not yet been tested for their performance in matrisome proteomics. We chose to test them on ovaries from women aged over 50 years, which could be considered the most challenging samples to use for extracting and solubilizing ECM proteins due to their high levels of collagen and fibrosis-like aspect [42]. With scarce samples of healthy ovarian biopsies, it can be difficult to obtain sufficient amounts of proteins to generate high-quality MS data. We therefore paid special attention to sampling standardization and screened different mechanical disruption lysis matrices to select the one that yielded the highest amounts of protein. This turned out to be the stainless-steel beads measuring 0.3 mm in diameter. This approach overcame ill-adapted, time-consuming, partial mechanical lysis and possible cross-contamination encountered when using other methods (e.g., pestle and grinder).

Protein extraction, including lysis of cells, release of proteins from different cell compartments, and solubilization of proteins into extraction buffer, is the first step in protein sample preparation. In this context, addition of 300 mM NaCl to HEPES buffer and its concomitant use with detergents proved to be effective in solubilizing mainly cellular proteins and enhancing detergent efficiency. Our approach, combining centrifugation and MS-compatible detergent along with LC-MS/MS successfully identified 131 matrisome proteins with good reproducibility, which is the highest number of ECM proteins to have ever been described in the human ovary thanks to the use of RP at 0.2% with simple 2-step fractionation [39]. Moreover, RP has a major advantage over its commercialized competitors, as its cleavage does not result in amine products following degradation (Supplementary Data S4). Our proposed method is therefore compatible with isobaric amine-reactive tandem mass tag labeling reagents, such as tandem mass tag (TMT) and isobaric tags for relative and absolute quantitation (iTRAQ) [16,26].

To avoid the above-mentioned caveats associated with chemical digestion of insoluble ECM pellets, we chose to use Liberase DH for pre-enzymatic digestion. Liberase DH is a mixture of highly purified collagenase I and II and neutral protease dispase produced successively by *Clostridium histolyticum* and *Bacillus polymyxa*, and formulated for efficient, gentle, and reproducible dissociation of tissue from a wide variety of sources [43]. While collagenase cleaves specifically helical regions in native collagen preferentially at the X-Gly bond in the sequence Pro-X-Gly-Pro, where X is most commonly a neutral amino acid, dispase cleaves peptide bonds containing leucine and phenylalanine (e.g., fibronectin). Use of Liberase DH decreased insoluble protein agglomeration and cleaved collagen as the most abundant substrate, allowing better detection of low abundance proteins. Moreover, Kuljanin et al. [44] already demonstrated that collagenase preferentially cleaves collagen, with minor off-target effects, and described the advantages of its use in proteomics.

In line with the literature, our previously published proteomic method using Liberase did not spot any interference with non-collagenous protein detection [39]. Following its use and upon double trypsin/Lys-C digestion, we did not observe any residual undigested material in our samples, which could easily be left behind and discarded in widely accepted proteomic methods.

Our study also revealed that a high percentage of ECM proteins could be lost, even in detergent-free buffers in intermediate fractions, which are not considered for analysis in most published ECM proteomic studies [15]. This observation is another key element behind our matrisome-tailored proteomic pipeline based on the principle of divide-and-conquer matrisome proteins (DC-MaP). While centrifugation enabled us to separate the soluble fraction from the insoluble fraction enriched with collagens and glycoproteins, pre-enzymatic Liberase DH digestion of the resulting pellet optimized identification of low-abundance matrisome-associated proteins entrapped in insoluble protein agglomerates. Only through combined analysis of both fractions could we capture the full picture of the ovarian matrisome in our samples without jeopardizing detection of soluble ECM-associated proteins that could easily be overlooked in standard enrichment procedures (Supplementary Data S3). Moreover, we foresee a possible application of our fractionation method in monitoring changes in solubility and crosslinking of ECM proteins, in different experimental conditions for example between healthy and fibrotic tissues or between normal and cancerous tissues [45,46,47].

Our comparison between 3- and 2-step fractionation demonstrated the superiority of the shorter procedure, leading us to conclude that more elaborate fractionation and multiple surface contact with the protein sample can lead to huge matrisome protein loss. Thus, DC-MaP is a combination of optimal extraction conditions for a high throughput matrisome proteomics designed to overcome encountered difficulties with conventional methods.

Our DC-MaP technique using 2-step fractionation with 0.2% RP complemented by insoluble pellet digestion with Liberase DH is the first validated application of an MS-compatible detergent in matrisome proteomics, representing a robust, affordable (Supplementary Data S5) and straightforward proteomic procedure tailored to matrisome protein discovery. Furthermore, our technique could be safely applied with isobaric amine-reactive tandem mass tag labeling reagents in comparative studies, since RP cleavage does not release primary amines (Supplementary Data S4). We believe the most accurate characterization of ECM proteins from different tissue types and even those with limited availability can be achieved with our DC-MaP method. This will help advance organ engineering efforts by generating a molecular readout that can be correlated with functional outcomes to drive the next generation of engineered organs (e.g., the artificial ovary) and fasten the transition of proteomics to clinical practice through enabling biomarker discovery at the level of ECM in an unbiased fashion.

## 4. Materials and Methods

### 4.1. Collection of Ovarian Tissue and Biopsy Processing

Use of human ovarian cortex was approved by the Institutional Review Board of the Université Catholique de Louvain on 13 May 2019 (IRB reference 2012/23MAR/125, registration number B403201213872). Ovarian biopsies were taken from adult patients (*n* = 6; mean age [±SD] = 58 ± 9 years) after obtaining their informed consent. All women were undergoing laparoscopic surgery for benign gynecological diseases not related to the ovaries. Biopsies were immediately transported on ice to the laboratory in minimal essential medium (MEM)-GlutaMAX (Gibco, Invitrogen, Merelbeeke, Belgium). Fresh biopsies were cut into fragments and cryopreserved [48]. On the day of biopsy processing for protein extraction, all samples were thawed [48] and cut into 3 × 3 × 1 mm fragments using graph paper. Excess medium was removed with gauze and 10 mg wet weight of each sample was processed as described below.

### 4.2. Selection of Lysing Matrix

Lysing matrices were used to supersede ill-adapted, time-consuming, partial mechanical lysis procedures and avoid possible cross-contamination encountered in other applied methods (e.g., pestle and grinder). Outcomes of five mechanical lysis conditions (Cd) using different bead types were compared: Cd1 (Full Moon, BioSystems, Sunnyvale, CA, USA), Cd2 (Precellys Lysing Kit MK28, Bertin Technologies, Aix en Provence, France), Cd3 (Lysing Matrix Z, MP Biomedicals, Illkirch-Graffenstaden, France), Cd4 (Lysing Matrix A, MP Biomedicals) and Cd5 (Lysing Matrix D, MP Biomedicals), which was preceded by a tissue chopping step (McIlwain Tissue Chopper, Mickle Laboratory, Guildford, UK).

A tissue fragment from each patient (*n* = 3/condition) was transferred to an empty 2 mL FastPrep^®^ Lysing matrix tube (MP Biomedicals) filled with one of the different bead types. As lysis buffer, we used a 25 mM HEPES solution (pH = 7.6) supplemented with protease inhibitors (1× complete tablet, 50 mM sodium fluoride [NaF], 0.25 mM sodium orthovanadate and 0.25 mM phenylmethylsulfonyl fluoride [PMSF]). Each 2 mL lysis tube was filled with 200 µL lysis buffer and homogenized at 4 °C with Precellys Evolution (Bertin Technologies) at the following settings: speed 9500 rpm, cycle 4 × 20 s, pause 15 s. After homogenization, the tubes were kept on ice until elution. The tubes’ bottoms were inverted and pierced with a clean needle, then the cap was slightly loosened and placed in a Falcon tube. The Falcon tubes were centrifuged at 3000× *g* for 5 min at 4 °C. Pass-throughs were subsequently transferred to 1.5 mL conical bottom centrifuge tubes and centrifuged again at 16,000× *g* for 30 min at 4 °C, before analyzing the supernatants. Comparisons between lysing matrices were based on the total amount of extracted soluble proteins, their quality, preservation of core matrisome proteins, and method reproducibility, as described below.

BCA assay (Thermo Scientific^TM^, San José, CA, USA) was used for quantification of total extracted soluble proteins. Their quality was monitored by SDS-PAGE using the Mix-n-Stain™ Total Protein Prestain Kit (Biotium, Fremont, CA, USA). Preservation of core matrisome proteins in the insoluble fraction was monitored in the supernatant by near-infrared western blot using two ECM antibodies, anti-osteoglycin (Invitrogen, Carlsbad, CA, USA) and anti-fibronectin (Abcam, Cambridge, MA, USA), and revealed by donkey anti-rabbit secondary antibody CF^®^770 (Biotium).

### 4.3. Formulation of Lysis Buffer

Starting from a limited sample size could be a burden to full matrisome detection. Therefore, it was essential to maximize the amount of total proteins from the beginning and wash out as many intracellular proteins as possible from the insoluble ECM to decrease the dynamic range and improve matrisome detection by MS. In this context, three concentrations of NaCl were used to supplement the HEPES lysis buffer (*n* = 3), 75 mM, 150 mM, and 300 mM, using the selected lysis matrix as described above. Supernatants were separated from the homogenates and the amount of total soluble proteins extracted with each NaCl concentration was evaluated by BCA assay (Thermo Scientific). Near-infrared western blot was used to evaluate the impact of each NaCl concentration on intracellular protein extraction, namely histone (nucleus) and actin (cytoskeleton) using anti-histone (Abcam) and anti-actin (Abcam) antibodies, respectively, before being revealed by donkey anti-rabbit secondary antibody CF^®^770 (Biotium).

### 4.4. Deciphering Matrisome Composition Using 2-Step Fractionation and MS-Compatible Detergents

#### 4.4.1. Sample Fractionation

Ovarian tissue samples (10 mg) were mechanically lysed using the selected lysing matrix. At this point, optimized lysis buffer was used to resuspend MS-compatible detergents, namely 3273, DDM, RP, and PM, to achieve 0.1%, 0.2%, and 0.5% concentrations. The homogenate was eluted with the needle technique (described above) by centrifugation at 3000× *g* for 5 min at 4 °C. The eluate was then centrifuged again at 16,000× *g* for 30 min at 4 °C to recover the supernatant labeled as “fraction 1” (soluble) and the remaining pellet obtained labeled as “fraction 2” (insoluble).

#### 4.4.2. Yield of Extracted Proteins

Total protein concentration measurement (BCA) was conducted on fraction 1 to evaluate the effect of detergent type and concentration on the yield of extracted proteins. To avoid possible interference of used detergents with BCA quantification, lysis solutions containing the detergents were used as blank.

#### 4.4.3. Western Blot

A total of 20 µg of fractions 1 and 2 obtained with each detergent were loaded onto 4–15% gradient bis-acrylamide gels (Biorad, Hercules, CA, USA), using *n* = 3 per condition for immunoblotting. Since fraction 2 is insoluble, it was first solubilized using 100 µL of 1% SDS and incubated for 1 h in the thermomixer at 60 °C under 1400 rpm agitation. Ceramic beads were added to the vial to improve protein solubilization. The obtained homogenate was centrifuged at 3000× *g* for 5 min. Another 100 µL of 1% SDS was added to the remaining insoluble pellet and incubated for 1 h in thermomixer at 37 °C under agitation at 1400 rpm. Both solubilized homogenates were pooled, spun, and quantified by BCA assay for SDS-PAGE. Electrophoresis migration was run at a voltage of 55 V for 30 min and 170 V for 1 h. An adapted Laemmli buffer formulation was used at 1× final concentration for protein denaturation: 0.44% [*wt/vol*] SDS, 12.4 mM Tris-HCl pH 6.8, 1% [*vol/vol*] 2-mercaptoethanol, 4% [*vol/vol*] glycerol, 0.00125% [wt/vol] bromophenol blue, supplemented with 100 mM dithiothreitol (DTT). Proteins were then transferred to immobilon-FL membrane (Biorad) in an 18% methanol transfer buffer and blocked with SuperBlock™ (TBS) Blocking Buffer (Thermo Scientific). The blocking solution was diluted 2× in 1× Tris-Buffered Saline and 0.1% Tween^®^ 20 Detergent (TBST) to dilute antibodies and secondary antibodies (Supplementary Data S2). Representative primary antibodies for each of the cellular (β1-integrin, histone, and actin) and ECM (osteoglycin and fibronectin) proteins were used to monitor protein fractionation. Following primary antibody incubation at 4 °C overnight under mild agitation, 6 repeated 5 min washes of the membrane were carried out before and after secondary antibody incubation (6 with TBST before incubation, and 3 with TBST followed by 3 with PBS after incubation). Each antibody was tested with a total protein extract obtained from a pool of ovarian tissue fragments from the group of patients whose tissue was solubilized similarly to fraction 2 before loading. This control sample was included in each western blot. To enable reliable quantification of the staining, all tested samples were also loaded in parallel SDS-PAGE gel and labeled with PageBlueTM protein staining (Thermo Scientific) to allow densitometry normalization per total loaded protein. All membranes were scanned with an infrared imaging system (Odyssey, Li-Cor Biosciences, Lincoln, NE, USA) and analyzed using Licor Image Studio Lite software (Li-Cor Biosciences).

### 4.5. Impact of a Third Fractionation Step on ECM Recovery

#### 4.5.1. Sample Fractionation

A supplemental experiment was conducted to check the reliability of the 2-step fractionation procedure, exclude the possibility that high salt (NaCl) levels in the lysis buffer might interfere with the action of the selected MS-compatible surfactant, and evaluate its impact on ECM detection. For this, ovarian fragments (*n* = 6) were first mechanically lysed in 200 µL of a detergent-free lysis buffer containing 25 mM HEPES solution (pH = 7.6) with 300 mM NaCl, supplemented with protease inhibitors (1× complete tablet, 50 mM NaF, 0.25 mM sodium orthovanadate and 0.25 mM PMSF). The previously selected lysis matrix was used following the same mechanical lysis settings. Eluted homogenates were centrifuged at 16,000× *g* for 30 min at 4 °C and the supernatants were labeled as “fraction 1” (soluble). The remaining pellets were resuspended in 60 µL MS-compatible detergent prepared in low-salt buffer containing 25 mM HEPES solution (pH = 7.6) and 75 mM NaCl with protease inhibitors. The samples were then sonicated for 30 min at 37 °C with regular vortexing every 5 min and centrifuged at 16,000× *g* for 30 min at 4 °C. The remaining supernatant formed “fraction 2” (soluble), while the final pellet constituted “fraction 3” (insoluble).

#### 4.5.2. Comparison of 2-Step and 3-Step Procedures

Qualitative and quantitative differences were evaluated between matrisome proteins detected in the 2- and 3-step procedures. For qualitative comparisons, we considered the type and category of matrisome proteins and the number of fractions that yielded the most complete description of the human ovarian matrisome. On the other hand, quantitative comparisons took into account the number and abundance of detected matrisome proteins and their recovery.

### 4.6. Sample Preparation for MS Analysis

Ovarian tissue fragments (*n* = 3) were mechanically lysed in the presence of MS-compatible detergents to obtain soluble and insoluble fractions by the 2-step and 3-step fractionations, as described above. Insoluble fractions were enzymatically digested using 1.35 mg/mL Liberase^TM^ DH (Roche Diagnostics, GmbH, Mannheim, Germany) in 100 mM triethylamonium bicarbonat (TEAB) and 5 mM calcium chloride (CaCl_2_). The samples were incubated at 37 °C for 2 h under agitation (140 rpm) with regular 30-s vortexing every 30 min. The enzymatic reaction was halted by the addition of 20 mM EDTA. BCA assay was used to quantify total proteins by subtracting Liberase^TM^ DH amounts from the insoluble fractions.

#### 4.6.1. Soluble Fraction Processing

Thirty µg of total soluble proteins were reduced with a 10 mM final concentration of DTT and incubated for 30 min at 56 °C, and subsequently alkylated with a 60 mM final concentration of chloroacetamide for 25 min at room temperature (RT) in the dark. The proteins were then precipitated using the methanol-chloroform method, before being resuspended in 100 mM TEAB (pH 8) and further digested with Lys-C/trypsin at an enzyme:substrate ratio of 1:25 [*wt/wt*] overnight at 37 °C. The reaction was halted by adding fresh TFA to a final concentration of 0.2% [*vol/vol*] (pH 2). The samples were then centrifuged at 16,000× *g* for 10 min at 4 °C. Recovered peptides were dried in a speedvac, resuspended in 50 µL of 3.5% acetonitrile (ACN) [*vol/vol*] and 0.1% TFA [vol/vol] in water, and quantified by Pierce™ Quantitative Colorimetric Peptide Assay (#23275, Thermo Scientific) according to the manufacturer’s instructions.

#### 4.6.2. Insoluble Fraction Processing

Fifty µg of total insoluble proteins were resuspended in a 6 M final concentration of urea in 100 mM TEAB, with continuous vortexing and sonication. Proteins were reduced in a 5 mM final concentration of DTT and incubated for 1 h at 37 °C. After cooling to RT, cysteines were alkylated by addition of 25 mM chloroacetamide for 30 min at RT in the dark. No precipitation was conducted during insoluble fraction processing to avoid resolubilization problems and to get advantage of the trypsin-enhancing action of MS-compatible detergents. Protein digestion was performed using Lys-C/trypsin at an enzyme:substrate ratio of 1:50 [*wt/wt*] and incubation for 2 h at 37 °C under 1000 rpm agitation. After reducing urea concentration to 1 M by addition of 100 mM TEAB, a second aliquot of trypsin was added to the sample at an enzyme:substrate ratio of 1:100 [*wt/wt*] and incubated overnight at 37 °C with continuous agitation at 1000 rpm. The reaction was halted by the addition of freshly prepared 50% TFA [*vol/vol*], pH 2. The acidified sample was centrifuged at 16,000× *g* for 10 min at RT to eliminate MS-compatible detergents. Digests were first desalted and concentrated on HyperSep C18 cartridges (50 mg/mL, Thermo Scientific, San Jose, CA, USA) according to the manufacturer’s instructions, and then dried using a speedvac. Peptides were resuspended in 50 µL of 3.5% ACN [*vol/vol*] and 0.1% TFA [*vol/vol*] in water and quantified by the Pierce™ Quantitative Colorimetric Peptide Assay following the manufacturer’s instructions.

#### 4.6.3. Liquid Chromatography-Tandem-Mass-Spectrometry (LC-MS/MS)

One µg of peptides dissolved in solvent A (0.1% TFA in 2% ACN) was directly loaded onto a reversed-phase pre-column (Acclaim PepMap 100, Thermo Scientific) and eluted in backflush mode. Peptide separation was achieved using a reversed-phase analytical column (Acclaim PepMap RSLC, 0.075 × 250 mm, Thermo Scientific) with a 140 min linear gradient of 4%–32% solvent B (0.1% TFA in 80% ACN) for 99 min, 32%–60% solvent B for 10 min, 60%–95% for 1 min and holding at 95% for the last 10 min at a constant flow rate of 300 nL/min on an Ultimate 3000 RSLN nanoHPLC system (Thermo Scientific). The peptides were analyzed by an Orbitrap Fusion Lumos tribrid mass spectrometer (Thermo Fisher Scientific) with enabled advanced peak determination (APD). The peptides were subjected to a nanospray ionization source, followed by MS/MS in the Fusion Lumos coupled online to the UPLC. Intact peptides were detected in the Orbitrap at a resolution of 120,000 and MS/MS spectra were acquired in the IT after HCD fragmentation at 30%. A data-dependent procedure of MS/MS scans was applied for the top precursor ions above a threshold ion count of 5.0 × 10^3^ in the MS survey scan with 30 s dynamic exclusion. The total cycle time was set to 4 s. MS1 spectra were obtained with an AGC target of 4 × 10^5^ ions and a maximum injection time of 50 ms. MS2 spectra were acquired with an AGC target of 1 × 10^4^ ions and a maximum injection time of 35 ms. For MS scans, the m/z scan range was 350 to 1800. MS/MS spectra were exported using the following settings: peptide mass range: 350–5000 Da, minimal total ion intensity: 500.

#### 4.6.4. Protein Identification and Quantification

Resulting MS/MS data were processed using Sequest HT search engine within Proteome Discoverer 2.3 against a human protein reference target-decoy database obtained from Uniprot (1 March 2020, 87,489 forward entries). Trypsin was specified as the cleavage enzyme, allowing up to 2 missed cleavages, 4 modifications per peptide, and up to 3 charges. Mass error was set to 10 ppm for precursor ions and 0.1 Da for fragment ions, and considered dynamic modifications were +15.99 Da for oxidized methionine and +57.00 for carbamidomethyl cysteine. The false discovery rate (FDR) was investigated using Percolator, and thresholds for protein, peptide, and modification sites were specified at 1%.

### 4.7. Data Mining and Data Visualization

Matrisome protein recognition in raw MS data was achieved by comparison of confidently detected proteins with the Matrisome Project data set [49], an in silico-identified ECM protein set founded on the characteristic domain-based organization of ECM proteins. JMP^®^ 14 was used to manage data, conduct correlations, and generate qualitative analysis graphs.

### 4.8. Experimental Design and Statistical Rationale

All experiments included *n* = 3 biological replicates of menopausal ovarian tissue per each tested condition, except in the 3-step fractionation where the number of replicates was elevated to *n* = 6. A threshold was maintained to pinpoint the most confidently detected proteins to consider in our interpretation of results. This threshold included proteins with an FDR 1% and unique peptides ≥ 2 for each sample. XICs were performed by label-free quantification using Proteome Discoverer 2.3 (Thermo Fisher Scientific) and were regarded as an indicator of protein abundance. Sampling rate normalization was applied to all our data (mean XICs/number of detected proteins). After confirming data normality, two-way ANOVA was used to first compare the overall performance of detergents irrespective of their used concentrations, and then the outcomes of the same detergent at different concentrations. To evaluate the reproducibility of each procedure between replicates, Pearson’s correlation was used to calculate the correlation coefficient, r. ICCs were also acquired between replicates to further evaluate the consistency of each method using IBM SPSS Statistics 24 software (IBM Corp, Armonk, NY, USA), as described by Richard Landers [50]. Briefly, ICC is measured on a scale of 0 to 1; 1 represents perfect consistency (reproducibility) with no measurement/technical error, while 0 indicates no reliability.

Total abundance of a protein was considered as the sum of its XICs in all fractions, and the percentage per fraction was accordingly calculated. Log2 fold change of XICs between fraction 2 per fraction 1 was used to identify the most enriched protein matrisome categories in each fraction. To evaluate the total amount of identified matrisome proteins in all fractions, we used quantile normalization of the XIC sum in all fractions, as well as Log10 transformation.

To compare the identification of matrisome proteins using DC-MaP with a fractionation-free technique, we applied a paired *t*-test (core matrisome) and Wilcoxon’s test (matrisome-associated) according to data distribution in each matrisome category. In this comparison, only proteins confidently identified with both techniques were considered.

### 4.9. Data Availability

The mass spectrometry proteomics data have been deposited to the ProteomeXchange Consortium via the PRIDE [51] partner repository with the dataset identifier PXD020823 (https://www.ebi.ac.uk/pride/archive/projects/PXD020823). XLS file with quantification data is provided in Supplementary Table S2.

## Supplementary Materials

Supplementary Materials can be found at https://www.mdpi.com/1422-0067/21/23/9141/s1.

## Abbreviations

ACN	Acetonitrile
APD	Advanced peak determination
Cd	Condition
CNBr	Cyanogen bromide
DC-MaP	Divide-and-conquer matrisome protein
DDM	*N*-dodecyl β-D-maltoside
DTT	Dithiothreitol
ECM	Extracellular matrix
FASP	Filter-aided sample preparation
FDR	False discovery rate
GdnHCl	Guanidinium chloride
ICC	Intra-class correlation coefficient
iTRAQ	Isobaric tags for relative and absolute quantitation
MEM	Minimal essential medium
NaCl	Sodium chloride
NaF	Sodium fluoride
NH_2_OH	Hydroxylamine
PM	ProteaseMax
PMSF	Phenylmethylsulfonyl fluoride
RP	RapiGest
RT	Room temperature
TMT	Tandem mass tag
