# Supplementary material for: Divide-and-Conquer Matrisome Protein (DC-MaP) Strategy: An MS-Friendly Approach to Proteomic Matrisome Characterization
Source: Int J Mol Sci. 2020 Nov 30;21(23):9141. doi: 10.3390/ijms21239141 (PMC7730167; doi:10.3390/ijms21239141)
Supplement: Supplementary file 1 [file ijms-21-09141-s001.zip › Supplemental Data/Supplementary Data.pdf]

Cellular component	Antibody	Firm	Reference	Dilution
Membrane	Integrin-β1*	Cell signaling	9699	1/500
Cytoskeleton	Actin*	Abcam	ab8227	1/500
Nucleus	Histone*	Millipore	09-838	1/1000
ECM	Fibronectin**	Abcam	ab2413	1/1000
	Mimecan (OGN)*	Thermo Fischer	PA5-48255	1/500

(*) Secondary antibody was used at 1/10 000
(**) Secondary antibody was used at 1/ 20 000

Supplemental Data S1. Used antibodies and incubation conditions.

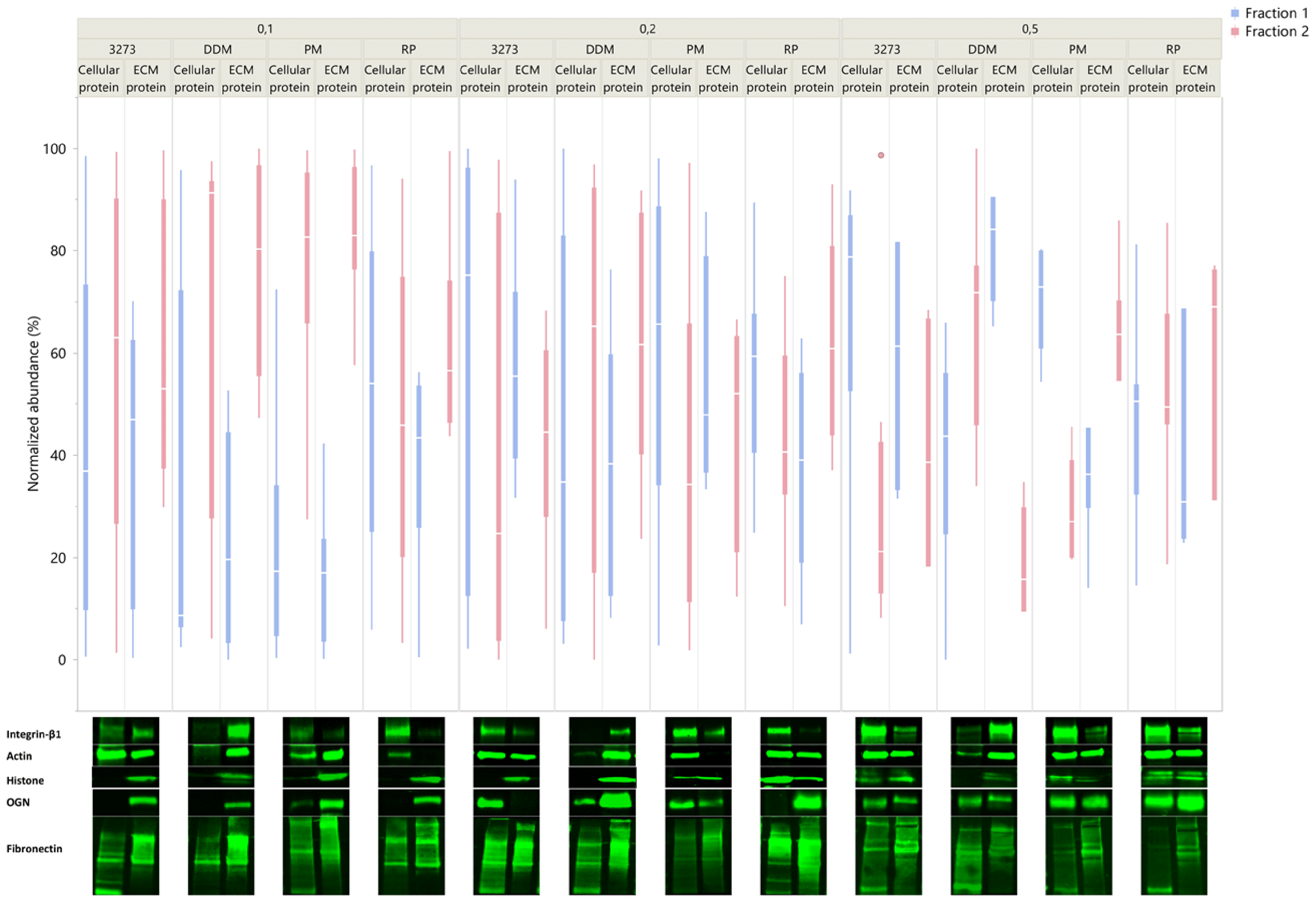

Supplemental Data S2. Immunoblotting. Densitometry results are expressed as a percentage of labeled proteins identified in each fraction. To enable reliable quantification of staining, all tested samples were also loaded in a parallel SDS-PAGE gel and labeled with PageBlueTM protein staining to allow densitometry normalization per total loaded protein.

A.

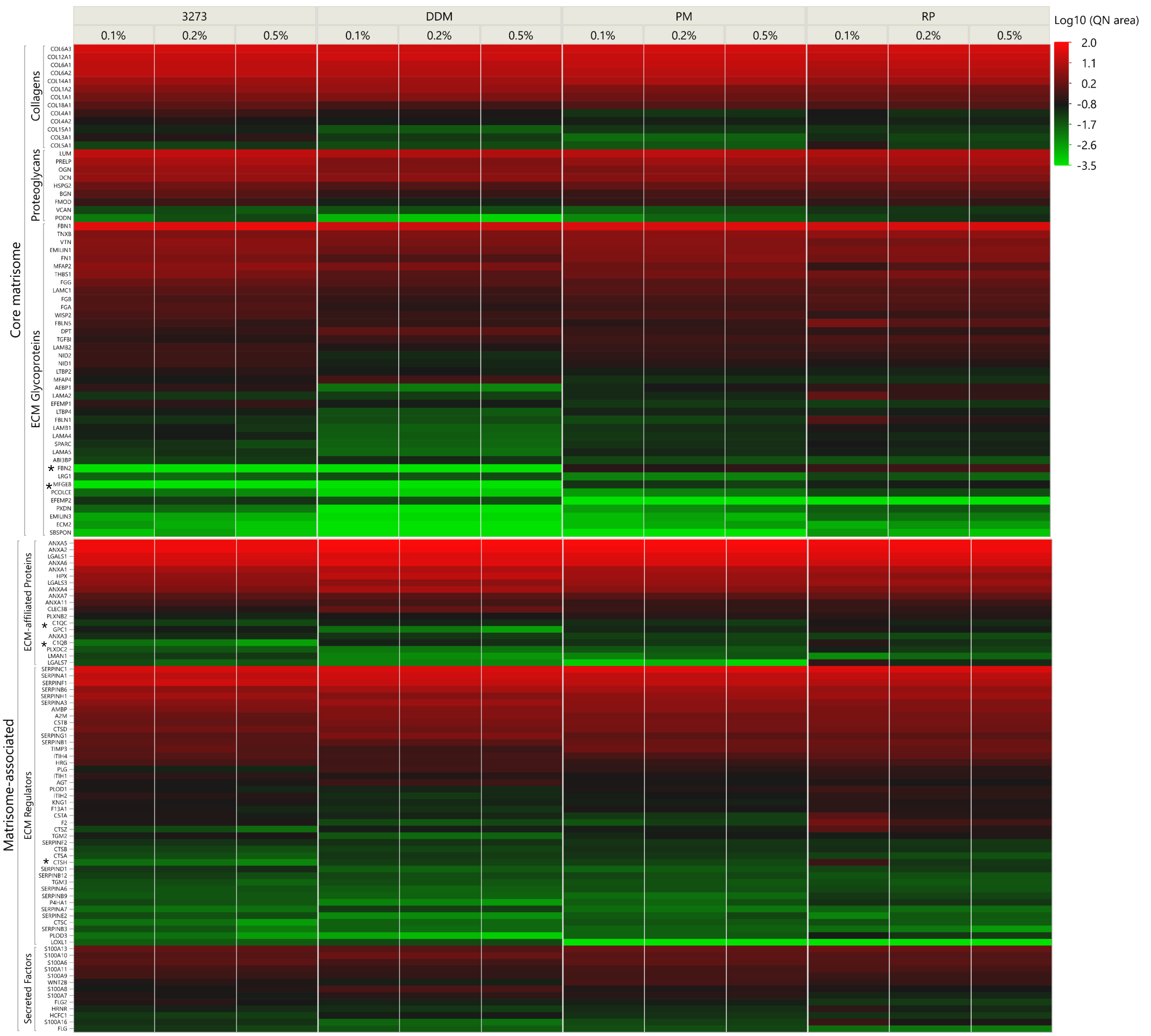
3273
DDM
PM
RP
0.1%
0.2%
0.5%
Log10 (QN area)
2.0
1.1
0.2
-0.8
-1.7
-2.6
-3.5
Core matrisome
Collagens
Proteoglycans
ECM Glycoproteins
Matrisome-associated
ECM-affiliated Proteins
ECM Regulators
Secreted Factors

B.

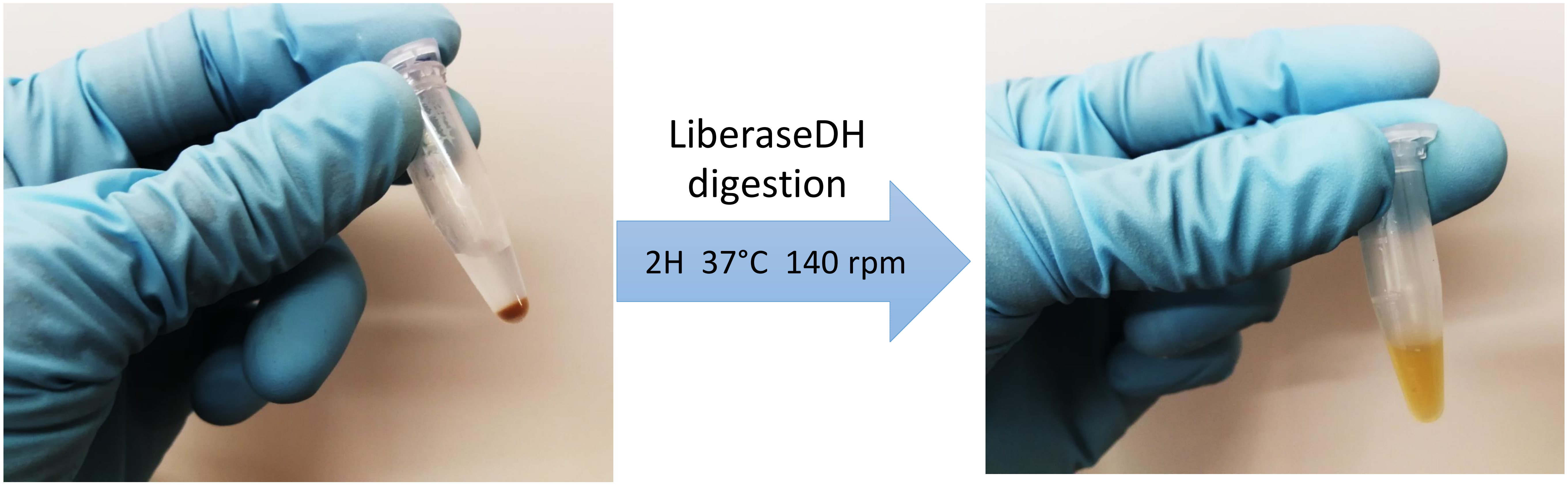
LiberaseDH
digestion
2H 37°C 140 rpm

Supplemental Data S3. Heat map of total matrisome protein abundance in fractions 1 and 2 combined. A) Protein abundance expressed in XICs was normalized by quantile normalization and transformed to Log10. B) Pictures of fraction 2 (insoluble pellet) before and after Liberase DH digestion. Proteins cited in the text are labeled with asterisks.

RP

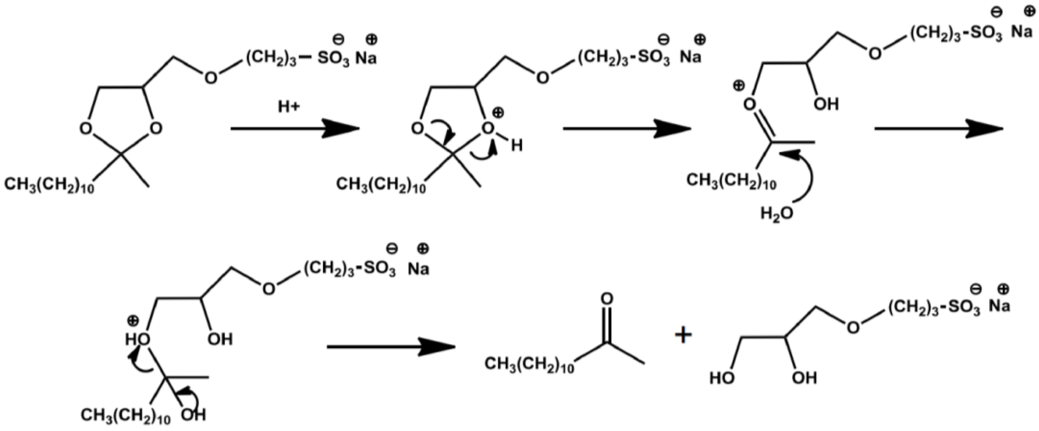

PM (X=O) vs 3273 (X=S)

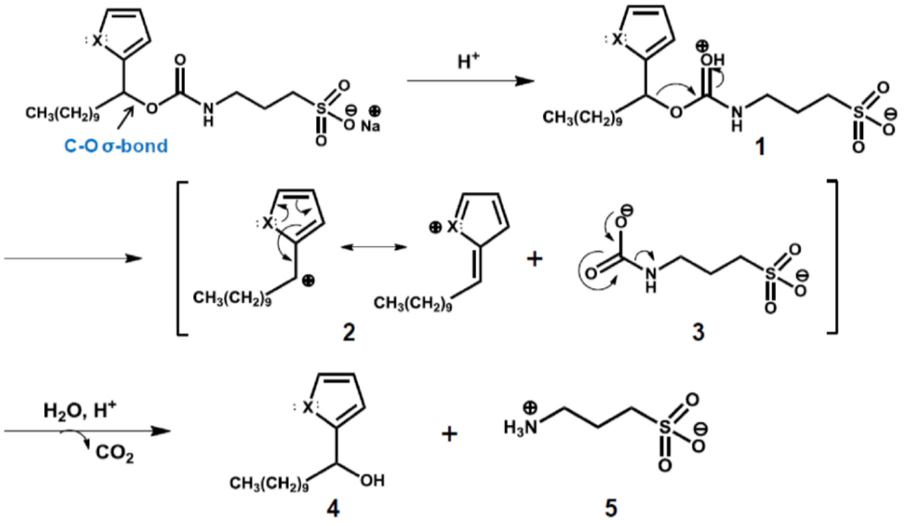

Supplemental Data S4. Degradation mechanism of MS-compatible surfactants. RP has a ketal functional group, which is susceptible to acid hydrolysis (pH<5). Acidification induces fragmentation of this functional group into a hydrophilic anionic molecule and a hydrophobic molecule ($CH_3(CH_2)_{10}COCH_3$). PM and 3273 have similar molecular structures that differ only in their labile groups, which contain a furan ring (X=O) and a thiophene ring (X=S) respectively. Generally, at pH<2, the carbonyl oxygen atom would

be protonated and lead to degradation of molecule 1. The furanyl group (X=O) is a better electron-donating group than the thiophene group (X=S) and, so cleavage of the C-O sigma bond will occur more quickly in PM. Subsequently, intermediate 3 undergoes decarboxylation (loss of carbon dioxide (CO_2)). This provides a strong driving force for degradation of the surfactant into CO_2 and a zwitterionic species (3-ammoniopropane-1-sulfonate) (molecule 5). Intermediate 2 then undergoes addition of water, producing an alcohol 4. It is therefore expected that PM will degrade more easily into CO_2, R-OH (molecule 4) and a small zwitterionic species (molecule 5) due to its furan ring compared to 3273, which contains a thiophene ring (10).

Surfactant	Type	CMC (%) T° 20-25°C
SDS	Anionic	0.030
3273	Anionic	0.007
DDM	Non-ionic	0.009
PM	Anionic	>0.1
RP	Anionic	0.064

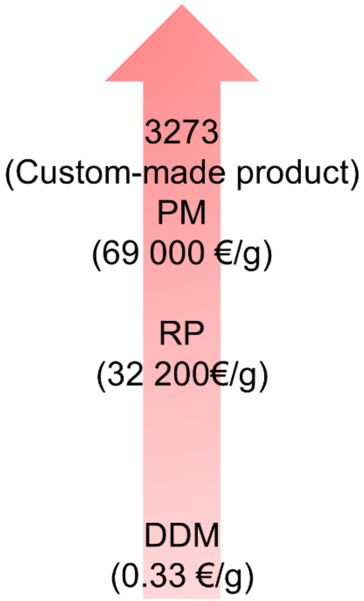

Supplemental Data S5. Surfactant properties and costs.

Supplemental Table S1. Table of results as a sum of all fractions.

Supplemental Table S2. Tables of protein quantification in all tested conditions.
